# Dietary exposure assessment and risk characterization of aflatoxin $\hbox {B}_1$ in cereal grains consumed in Somalia

**DOI:** 10.1038/s41598-026-37589-6

**Published:** 2026-01-28

**Authors:** Mohamed Aden Hersi, Osman Abubakar Fiidow

**Affiliations:** 1 Department of Agriculture, Faculty of Agriculture and Environmental Sciences, Somali National University, Mogadishu, Banadir Somalia; 2 Department of public health, Faculty of Health Science, Salaam University, Teleh Campus, Hodan District, Mogadishu, Banadir Somalia

**Keywords:** Aflatoxin B1, Dietary exposure, Cereals, Food safety, Risk characterization, Exposure assessment, Diseases, Environmental sciences, Health care, Risk factors

## Abstract

Aflatoxin $\hbox {B}_1$ (AFB$_1$) is a genotoxic carcinogen that frequently contaminates cereals in tropical food systems. Somalia lacks nationally representative dietary intake data to support exposure assessment and risk management. To estimate dietary exposure to AFB$_1$ from maize and sorghum among Somali adults and children and to characterize risk using internationally endorsed toxicological reference points. A deterministic exposure assessment integrated AFB$_1$ occurrence data from Somali market surveys with per-capita daily cereal intake derived from SIHBS 2022. Average Probable Daily Intake (APDI; ng/kg bw/day) was calculated for adults (60 kg) and children (20 kg) across mean, P50, P95, and P97.5 consumption levels combined with minimum, median, and maximum contamination scenarios. Margin of Exposure (MOE) was computed using BMDL$_{10}$ = 400 ng/kg bw/day. Maize—especially white maize—dominated exposure. Under median contamination, adult maize APDI ranged 293–1366 ng/kg bw/day (MOE 1.37–0.29), while children’s APDI ranged 878–4101 ng/kg bw/day (MOE 0.46–0.10). Sorghum generally contributed much lower exposure except under maximum contamination for red sorghum. Children had approximately three-fold higher exposure than adults on a body-weight basis. Estimated MOEs were far below 10,000, indicating very high priority for risk management. Strengthened regulation, monitoring, and post-harvest interventions are urgently needed, alongside improved dietary data systems.

## Introduction

Aflatoxins, particularly aflatoxin B_1_ (AFB_1_), are highly toxic secondary metabolites mainly produced by *Aspergillus flavus* and *Aspergillus parasiticus*. These compounds frequently contaminate staple crops such as maize, sorghum, and wheat, especially in warm and humid regions that favor fungal growth^1,2^. AFB_1_ is the most potent of the aflatoxins and has been classified by the International Agency for Research on Cancer (IARC) as a Group 1 human carcinogen, strongly associated with hepatocellular carcinoma^3^. In addition to carcinogenicity, chronic aflatoxin exposure contributes to acute liver toxicity, immune suppression, growth impairment in children, and heightened susceptibility to infectious diseases, making it a global public health challenge^4–6^.

At the global level, aflatoxins are consistently recognized as a leading food safety hazard, particularly in low- and middle-income countries (LMICs). Their occurrence is influenced by climatic factors, with climate change projected to expand aflatoxin-prone regions and intensify contamination risks^7–9^. International risk assessments by the Joint FAO/WHO Expert Committee on Food Additives (JECFA) and the European Food Safety Authority (EFSA) emphasize that AFB_1_ is a genotoxic carcinogen for which no safe threshold can be established, reinforcing the need for continuous exposure minimization^10,11^.

In sub-Saharan Africa (SSA), aflatoxin contamination of cereals, groundnuts, and milk is a pervasive food safety problem, with frequent exceedances of Codex Alimentarius and EU maximum limits^12–14^. Multi-country studies have reported high contamination levels in maize and sorghum from Nigeria, Ghana, Kenya, and Ethiopia, with exposure assessments showing margins of exposure (MOEs) well below international safety benchmarks^2,15,16^. Somalia faces a similar, if not more severe, challenge. Multi-mycotoxin surveys have documented AFB_1_ concentrations in Somali maize as high as 900 $\upmu$g/kg, far above Codex maximum levels^17^.

A critical barrier to evidence-based policymaking in Somalia is the absence of nationally representative dietary intake surveys. In such data-scarce environments, household expenditure surveys provide a feasible proxy for food consumption estimates, a method already applied in exposure assessments in other SSA countries^18–20^. These approaches, while imperfect, enable initial national-level exposure and risk characterization that can guide policy and investment in food safety systems.

This study aims to assess dietary exposure and characterize the health risk posed by AFB_1_ in cereals commonly consumed in Somalia, using deterministic models recommended by JECFA and EFSA. The analysis incorporates multiple exposure scenarios based on contamination levels and estimated intake to support evidence-based risk management, as well as inform future policy decisions and strengthen Somalia’s food safety framework.

## Materials and methods

### Study design and deterministic exposure framework

This study conducted a deterministic dietary exposure assessment of aflatoxin $\hbox {B}_1$ (AFB$_1$) among the Somali population by combining nationally representative consumption data from the Somalia Integrated Household Budget Survey (SIHBS 2022)^21^ with cereal contamination occurrence data reported by^17^. Deterministic approaches use fixed consumption and contamination inputs to derive the Average Probable Daily Intake (APDI) and are appropriate where full distributional data are unavailable. This deterministic exposure assessment and application of the Margin of Exposure (MOE) framework are consistent with international guidance from the European Food Safety Authority and the FAO/WHO Joint Expert Committee on Food Additives for the risk characterization of genotoxic and carcinogenic food contaminants, including aflatoxin $\hbox {B}_1$^10,11^.

### Consumption data and processing

Weekly household consumption quantities of maize grain and sorghum grain were extracted from the SIHBS 2022 microdata available on the NBS website. Consumption percentiles—mean, median (P50), 95th percentile (P95), and 97.5th percentile (P97.5)—were calculated among households reporting consumption of each cereal. Per-capita daily intake (g/person/day) was computed as:

1 $$\begin{aligned} \text {Daily intake (g/person/day)}=\frac{\text {Household 7-day intake (g)}}{\text {Household size}\times 7}. \end{aligned}$$

An average household size of six persons was used consistent with SIHBS 2022 (Table 1).

**Table 1:** Weekly household consumption of maize and sorghum.

Cereal	N (HH)	Consumption (g/household/week)				
		Mean	SD	P50	P95	P97.5
Maize	1615	1996	1672	1750	5000	7000
Sorghum	1257	2020	1811	1500	5400	7000

### Occurrence data

AFB$_1$ contamination levels for cereals were taken from^17^, which quantified AFB$_1$ in staple cereals sampled from Somali markets. Contamination data were available as minimum, median, and maximum concentrations ($\upmu$g/kg) for each cereal variety. Exposure was calculated separately for each variety to maintain transparency and avoid unverified weighting assumptions. Table 2 summarises the contamination levels used for exposure calculations.

**Table 2:** AFB$_1$ contamination levels ($\upmu$g/kg) reported by Wielogorska et al. (2019) and used for exposure assessment.

Cereal variety	Median ($\upmu$g/kg)	Minimum ($\upmu$g/kg)	Maximum ($\upmu$g/kg)
White maize	492	186	908
Yellow maize	162	25.5	781
White sorghum	0.9	0.67	3.2
Red sorghum	1.1	0.6	105

### Toxicological reference points

To contextualize exposure in terms of potential health impact, average body weights were set at 60 kg for adults and 20 kg for children, aligning with WHO regional reference values for African populations. For risk characterization, the benchmark dose lower confidence limit associated with a 10% increase in liver cancer incidence (BMDL_10_) was fixed at 400 ng/kg bw/day. This toxicological reference point has been endorsed by both JECFA^10^ and EFSA^11^ and serves as a critical threshold for evaluating population-level health risks.

### Exposure and risk calculations

Dietary exposure was quantified as the Average Probable Daily Intake (APDI), expressed in ng/kg bw/day, using the formula:

2 $$\begin{aligned} \text {APDI (ng/kg bw/day)} = \frac{\text {Contamination level (ng/g)} \times \text {Daily intake (g/day)}}{\text {Body weight (kg)}}. \end{aligned}$$

Risk characterization was performed using the Margin of Exposure (MOE), calculated as:

3 $$\begin{aligned} \text {MOE} = \frac{\text {BMDL}_{10}}{\text {APDI}}. \end{aligned}$$

Three exposure scenarios were considered for each cereal type and population group, based on mean, 95th percentile, and 97.5th percentile contamination levels. Lower MOE values indicate greater potential health concern, with values below 10,000 regarded as insufficiently protective by both EFSA^11^ and JECFA^10^. This combined approach of deterministic modeling, conservative assumptions, and internationally recognized toxicological benchmarks provides a transparent and scientifically robust framework for characterizing public health risks associated with aflatoxin B_1_ exposure in Somalia. High-percentile consumption scenarios (P95 and P97.5) are routinely applied in deterministic dietary exposure assessments to represent upper-bound intake conditions among high consumers, consistent with EFSA and FAO/WHO–JECFA guidance.

## Results and discussion

### Dietary exposure estimates

This study represents the first nationally representative dietary exposure assessment of aflatoxin $\hbox {B}_1$ (AFB$_1$) in Somalia, integrating occurrence data from Wielogorska et al. (2019) with nationally representative household consumption estimates derived from SIHBS 2022. Estimated dietary exposure was expressed as Estimated (Average Probable) Daily Intake (EDI/APDI) and Margin of Exposure (MOE), disaggregated by population group (adults and children) and consumption level (mean, P50, P95, and P97.5).

Across all exposure scenarios (Table 3), maize, particularly white maize, was the dominant contributor to AFB$_1$ exposure, reflecting its exceptionally high median contamination levels. For adults, median-occurrence white maize APDIs ranged from 390 ng/kg bw/day (mean intake) to 1366 ng/kg bw/day (P97.5 intake), while corresponding values for children ranged from 1169 to 4101 ng/kg bw/day. Children consistently exhibited approximately three-fold higher exposure than adults on a body-weight basis, attributable to lower body weight rather than higher absolute consumption. Sorghum generally resulted in substantially lower APDIs under minimum and median contamination scenarios; however, the maximum contamination scenario for red sorghum produced high exposure estimates, indicating the potential for sporadic contamination events.

**Table 3:** Estimated daily intake (EDI/APDI) of aflatoxin $\hbox {B}_1$ ($\hbox {AFB}_1$) in adults and children (ng/kg bw/day).

Variety	Scenario (C, $\upmu$g/kg)	Adults				Children			
		Mean	P50	P95	P97.5	Mean	P50	P95	P97.5
White maize	Min (186)	147	111	369	517	442	332	1107	1550
White maize	Median (492)	390	293	976	1366	1169	878	2927	4101
White maize	Max (908)	719	540	1801	2523	2156	1621	5403	7568
Yellow maize	Min (25.5)	20.20	15.15	50.58	70.85	60.60	45.55	151.74	212.55
Yellow maize	Median (162)	128	96	321	450	385	289	964	1350
Yellow maize	Max (781)	618	465	1548	2167	1855	1394	4647	6510
White sorghum	Min (0.67)	0.54	0.40	1.44	1.86	1.61	1.20	4.31	5.58
White sorghum	Median (0.9)	0.72	0.54	1.93	2.50	2.17	1.61	5.79	7.50
White sorghum	Max (3.2)	2.57	1.90	6.86	8.89	7.69	5.71	20	27
Red sorghum	Min (0.6)	0.48	0.36	1.29	1.67	1.44	1.07	3.86	5.00
Red sorghum	Median (1.1)	0.88	0.66	2.36	3.06	2.65	1.96	7.07	9.17
Red sorghum	Max (105)	84	62	225	292	252	187	675	875

### Exposure patterns and risk levels

Table 4 summarises the MOE values for adults and children under different consumption and contamination scenarios. MOE values were consistently far below 10,000 for maize across consumption percentiles in both adults and children, indicating a high priority for risk management under internationally accepted approaches for genotoxic carcinogens. Sorghum varieties generally produced substantially higher MOEs under minimum and median contamination scenarios, suggesting comparatively lower relative concern; nevertheless, maximum contamination scenarios for red sorghum resulted in markedly reduced MOEs, highlighting the importance of addressing contamination hotspots.

For children, exposure levels were approximately 3–4 times higher than for adults due to lower body weight, with MOE values critically low—especially for maize (white and yellow), where MOE reached 0.05 and 0.06 at P97.5, respectively (Table 4). MOE values were far below 10,000, a reference value used to indicate low concern for genotoxic carcinogens; therefore, these results indicate a high priority for risk management (EFSA, 2020; JECFA, 2018).

**Table 4:** Margin of exposure (MOE) for aflatoxin $\hbox {B}_1$ (AFB$_1$) in adults and children (BMDL$_{10}$ = 0.4 $\mu$g/kg bw/day).

Variety	Scenario	Adults				Children			
		Mean	P50	P95	P97.5	Mean	P50	P95	P97.5
White maize	Min	2.72	3.61	1.08	0.77	0.91	1.21	0.36	0.26
White maize	Median	1.03	1.37	0.41	0.29	0.34	0.46	0.14	0.10
White maize	Max	0.56	0.74	0.22	0.16	0.19	0.25	0.07	0.05
Yellow maize	Min	19.80	26.38	7.91	5.65	6.61	8.79	2.64	1.88
Yellow maize	Median	3.12	4.15	1.25	0.89	1.04	1.38	0.42	0.30
Yellow maize	Max	0.65	0.86	0.26	0.18	0.22	0.29	0.09	0.06
White sorghum	Min	745	1003	279	215	248	334	93	71
White sorghum	Median	554	747	207	160	185	249	69	53
White sorghum	Max	156	210	58	45	52	70	19	15
Red sorghum	Min	832	1120	311	240	277	373	104	80
Red sorghum	Median	454	611	170	131	151	204	57	44
Red sorghum	Max	4.76	6.41	1.78	1.37	1.58	2.13	0.59	0.46

### Regional and global comparison

The exposure estimates reported here are comparable to, and in several cases exceed, those reported in other high-exposure settings in sub-Saharan Africa. For example, maize-based AFB$_1$ intakes in Nigeria ranging from 150–500 ng/kg bw/day have been reported by^22^, while^23^documented low MOEs in Kenyan populations consuming contaminated maize.^2,24^ similarly reported widespread aflatoxin exposure in West Africa, particularly among children. More recent syntheses by^25^indicate that MOE values below 100–and in some cases below 10–are common in African dietary exposure assessments, and^26^ documented excessive exposure among infants and young children in East Africa. Taken together, the Somali exposure estimates are at least as severe as those reported elsewhere in the region and may be more concerning given limited systematic monitoring.

### Public health implications

AFB$_1$ is classified as a Group 1 carcinogen by the International Agency for Research on Cancer^3^, with a well-established causal link to hepatocellular carcinoma. Chronic dietary exposure is of particular concern in populations with hepatitis B or C co-infection, which synergistically increases liver cancer risk^27^. Beyond carcinogenicity, aflatoxin exposure has been associated with immune suppression, impaired child growth, reduced vaccine efficacy, and increased susceptibility to infectious disease^25,28–30^. Xia et al.^31^ reported that elevated aflatoxin biomarker levels in acutely ill children were significantly associated with increased mortality, underscoring broader health implications of chronic exposure. In Somalia, where cancer surveillance is limited and child undernutrition remains widespread, aflatoxin exposure likely represents a substantial but under-recognized public health burden.

### Trade, food safety, and economic implications

From a trade perspective, Somalia faces substantial risks. The European Union limit for AFB$_1$ in cereals is 2 ng/g, far below observed contamination levels. This effectively excludes Somali cereals from high-value export markets, as similar contamination issues have led to frequent rejections of African commodities^32^.

From a domestic food safety perspective, the same contamination levels imply that cereals entering local markets may frequently exceed health-based risk-management benchmarks, underscoring the need for enforceable limits, routine surveillance, and post-harvest control measures. Regionally, aflatoxin contamination has been identified as one of the major non-tariff barriers to intra-African trade, especially for maize^33,34^.

### Uncertainty and limitations

This assessment is subject to several sources of uncertainty. First, AFB$_1$ occurrence data were available only as minimum, median, and maximum values rather than full concentration distributions, which precluded probabilistic modelling. Second, dietary intake was estimated from household-level SIHBS data rather than individual recalls, and consumption percentiles were calculated among consuming households only, potentially overestimating exposure for the general population. Third, contamination data (2019) and consumption data (2022) were collected in different years, and changes in climate, storage practices, or cereal sourcing over time may influence current exposure levels.

To address these constraints transparently, we applied a scenario-based deterministic approach combining multiple contamination scenarios with several consumption percentiles to bracket plausible exposure ranges, consistent with EFSA and FAO/WHO–JECFA guidance. Margin of Exposure values are therefore used to support risk prioritisation rather than to quantify absolute cancer risk. Despite these limitations, the analysis provides the first nationally representative baseline for aflatoxin $\hbox {B}_1$ exposure in Somalia and identifies clear priorities for risk management and future data collection.

## Conclusion and recommendations

### Conclusion

This study presents the first national-level dietary exposure and risk characterization of aflatoxin $\hbox {B}_1$ in Somalia, focusing on maize and sorghum as staple cereals. By integrating mycotoxin occurrence data with nationally representative household consumption data, the study applied deterministic modelling to estimate EDI/APDI and MOE across multiple exposure scenarios. Results demonstrate consistently low MOE values, particularly for children consuming maize, indicating a high priority for risk management under internationally accepted frameworks. The study also illustrates the utility of household budget surveys as a pragmatic tool for exposure assessment in data-scarce settings, while acknowledging the limitations inherent in summary occurrence data and temporal mismatches.

### Recommendations

To address the significant dietary exposure to aflatoxin $\hbox {B}_1$ identified in this study, Somalia should strengthen its national food safety framework with a combination of regulatory, institutional, and community-level actions. Establishing clear and enforceable aflatoxin standards for cereals and cereal products, along with routine monitoring and laboratory capacity to support testing, will help ensure that contaminated grain is identified and managed before it enters the food supply. These regulatory efforts should be supported by practical systems for sampling, testing, and compliance that are tailored to the realities of local markets and storage conditions. At the same time, risk communication and education campaigns for farmers, traders, and consumers can promote simple, feasible practices, such as timely drying, proper storage, and sorting out damaged grain, that reduce contamination and exposure at the household and market levels.

Simultaneously, investment in improved post-harvest infrastructure and practices, together with incentives for producing and trading higher-quality grain, can reduce the prevalence of contamination across value chains. Strengthening coordination across agriculture, public health, standards, and trade sectors will support integrated policies that protect consumers while enhancing market opportunities. Finally, efforts to gather more detailed individual dietary intake data and targeted biomonitoring will refine future exposure assessments, improve risk prioritization, and help track the impact of interventions over time. These steps, taken together, can form a coherent strategy for reducing the public health burden of aflatoxin exposure in Somalia.

## Data Availability

The aflatoxin B1 occurrence data used in this analysis come from a previously published, peer-reviewed study and can be accessed directly in that article see [17]. Dietary consumption data were taken from the Somalia Integrated Household Budget Survey [21] carried out by the Somali National Bureau of Statistics (SNBS). Because SNBS controls access to the SIHBS microdata, these records are not publicly downloadable; however, they can be obtained by submitting a reasonable request and receiving approval through SNBS’s official microdata access process. Any results generated from these inputs in the present study including exposure estimates and risk metrics such as EDI/APDI and MOE summaries can also be requested from the corresponding author.
